# Case of Gun Shot Wound,—Death

**Published:** 1851-01

**Authors:** William Hunt

**Affiliations:** Resident Surgeon of the Pennsylvania Hospital


*Case of Gun Shot Wound,—Death.* Reported by William Hunt, M. D., Resident Surgeon of the Pennsylvania Hospital.

John Farley, aged 11. Was admitted into the Pennsylvania Hospital on the evening of April 18th, 1850. The person who brought him stated that he was shot through the hand, during a firemen's riot in Moyamensing. Such was found to be the case; a ball had passed through the left hand, between the heads of the first and second metacarpal bones: but the constitutional symptoms were so severe, that this wound alone was not sufficient to account for them; he was cold, exceedingly prostrate, and alarmed, and had a quick and almost imperceptible pulse. On further examination it was found that he was also shot through the left shoulder, the ball having fractured the head of the humerus and opened the joint. The ball could not be felt by the probe, which might have readily been passed into the wound further than was considered prudent. The patient was placed in bed, a piece of dry lint was applied to the wound, heaters were put to the feet, and sinapisms were ordered to the stomach and extremities; small quantities of wine and water were given at short intervals.

On uncovering the boy, to apply the mustard, it was noticed that the penis was gradually assuming the state of priapism, which soon became complete; this was followed by perfect paralysis of the lower extremities, both of sensation and motion.

These symptoms induced a close inspection of the back; nothing could be seen or felt of a foreign body, nor was there any other external wound than those mentioned. There was no paralysis of the upper extremities. The diagnosis, founded on these facts, was, that the ball had passed through the shoulder into the thorax, and had then travelled along its posterior parietes as far as the vertebral column, which was fractured at a point below where the brachial nerves are given off: the prognosis of course was fatal.

Carbonate of ammonia and brandy were given, in small doses, every half hour, until three doses were taken.

11 o'clock, P. M. Saw patient, with Dr. Peace. Symptoms not improved. Again examined the back and shoulders closely, but could detect nothing.

Quarter to 12. Little warmer; pulse still very feeble, not to be counted. On auscultation, found respiration very quick and loud, and a distinct friction sound was heard on the left side; paralysis and priapism continue. The patient is, and has been, perfectly conscious; complains none of pain, excepting when the wounded arm is disturbed.

1 o'clock. Warmer; pulse fuller; has passed a small quantity of blood from the mouth; some tendency to delirium. Expressed a desire to pass his water; a small catheter was introduced into the bladder, very little urine followed; paralysis and priapism continue, although latter not so perfect as before. Directed the patient to be kept quiet, and to take a small quantity of wine and water two or three times during the night.

16th, 7 A. M. Skin hot; tongue furred; respiration hurried and feeble; pulse full, very quick; paralysis and priapism as before; consciousness perfect.

9 o'clock. Skin hot; pulse 130; respiration 40, is feeble on the left side; cardiac sounds heard more distinctly to the right of sternum; no pain; drew off a small quantity of water by catheter. At 10, ordered an injection of oleum ricini, f.℥i., with molasses and water, Oss.

5 P. M. Complains of some pain in the breast, none in wound; drew half a pint of water from the bladder, high colored; bowels had been opened by the injection.

At 7. The abdomen was very tympanitic. ℞. Oleum Terebinth. f.℥ij., aquæ Oss., inject.

17th. Has had a restless night, been somewhat delirious; paralysis and priapism continue; abdomen less tense since the injection; pulse 130; tongue moist. Complains only of pain over front of chest; respiration labored; had five dry cups applied over chest, with some relief; obliged to draw off his urine twice daily; wound discharges very little; no swelling about the shoulder.

18th. Better, as regards respiration; pulse 120; urine plentifully secreted; bowels opened by injections; other symptoms the same.

19th. Better; pulse 112; tongue moist, slightly coated; respiration easier; secretion of urine very copious; obliged to introduce catheter four or five times daily; the penis is more rigid when the bladder is full. The patient never expresses a desire to urinate when alone, but he has frequently wished to do so while the water was flowing in a full stream from the instrument, of which he appears to be wholly unconscious.

3 P. M. Weaker; pulse very feeble, 120. Gave f.℥ss. of wine whey every two hours; this, at 8 o'clock, appears to have made him stronger; he ate a piece of toast with appetite, and was quite lively.

20th. Weaker; pulse 120; has involuntary discharges from the rectum. The urine is now loaded with a ropy mucus, and gives out a strong ammoniacal odor; not so plenty as before. Wound in shoulder discharges a light sanious liquid; no abatement in the priapism or paralysis.

21st. Continues as yesterday in every respect, but as to the urine, which now dribbles from him involuntarily.

22d. About the same. No pus is discharged from the wound; pulse 140.

23d, A. M. Pulse 120; says he feels well; priapism appears to have somewhat abated. P. M. Much worse; abdomen very tumid; priapism again perfect; urine still dribbles; pulse 166; respiration 50; tongue moist and furred; wound discharges but little of a light sanious liquid. ℞. Oleum Terebinth. f.ʒi, aquæ Oss. inject.

24th. Better; bowels had been opened; pulse 140; tongue clean, moist; appetite good; no swelling about the shoulder.

25th. Has remained all day without change; examined the back this morning. There is much more protuberance of the left inferior part of the thorax than the right; percussion dull, and respiration heard very indistinctly on that side.

26th. Much edema of prepuce; priapism still perfect; otherwise the same.

27th. The edema of prepuce is so great as to produce paraphymosis; this cannot be reduced, on account of the erect state of the penis. It was freely punctured, and the swelling was much diminished by night; there is no sensation in the organ.

28th. Same.

On the first of May the writer was obliged to leave the Hospital on account of sickness. The patient remained about the same until the 3d, when he sank rapidly, and died in the evening; the priapism continued until the day of his death. Consciousness remained perfect.

The post mortem examination (of which the writer is unable to obtain any full notes) showed that the ball had passed through the shoulder, producing a compound comminuted fracture of the humerus, just below its head, and had opened the joint; it then entered the thorax between the 2d and 3d ribs, and passed obliquely downwards through the pleura and posterior part of the lung, then through the body of the 5th or 6th dorsal vertebre, which was fractured completely through. The ball was found lodged in the spinal canal, at a point corresponding to the fracture.

The spinal marrow was softened, to a considerable extent, above and below the wound. The left lung was hepatised, and there was a large effusion of pus and bloody serum into the pleural cavity.

